# Spanish validation of the Pediatric Quality of Life Inventory (PedsQL™ 4.0) for parent report for toddlers (ages 2–4)

**DOI:** 10.1186/s12955-023-02128-8

**Published:** 2023-05-18

**Authors:** Melissa Liher Martínez-Shaw, Francisco Javier del Río, Yolanda Sánchez-Sandoval

**Affiliations:** 1 Biomedical Research and Innovation Institute of Cadiz, INIBICA, Cádiz, Spain; 2 Department of Psychology, Faculty of Educational Sciences, University of Cádiz, Puerto Real, Avenida República Saharaui, s/n, 11519 Spain

**Keywords:** Health-related quality of life, Instrumental study, Parents, Toddlers

## Abstract

**Background/objective:**

Health-related quality of life is a concept that includes aspects about physical, emotional and social well-being. The aim of the study was to validate the PedsQL for parent report for toddlers in Spain and provide reference data in a Spanish population.

**Method:**

The sample included 478 parents (89.5% mothers) of children aged 18–36 months (*M* = 26.75 months). Sociodemographic data were gathered, and the PedsQL and Kiddy-KINDL-R were completed by the participants.

**Results:**

The fit of the original structure of the PedsQL was acceptable (CFI = 0.93; TLI = 0.92; RMSEA = 0.06), and the results showed good internal consistency (*α* = 0.85). The items about nursery school were excluded, since not all the toddlers attended this type of educational centre. Significant differences were found in physical health and activities and in the total mean in terms of parent education level, and in social activities regarding gender. For the normative interpretation of the PedsQL, the first, second and third quartiles corresponded to 77.78, 84.72 and 90.28, respectively.

**Conclusions:**

This instrument is not only useful to individually evaluate the quality of life of a child with respect to his/her group, but also to measure the efficacy of a possible intervention.

Health-related quality of life (HRQOL) is a concept associated with the health aspects of quality of life, including physical, emotional and social well-being. It is commonly perceived as a multidimensional, dynamic and subjective idea. Generally, it considers the impact of disease and treatment on daily functioning and disability. It has also been measured to express the effect of health perception on a skill to have a satisfying life [1, 2]. One of the most frequently used HRQOL models is the one developed by Wilson and Cleary [2] [3], which links biological and physiological variables to subjective health constructs in order to measure HRQOL.

Traditionally, quality of life has been studied in adulthood due to its subjective and self-reported nature. However, in recent decades, there has been a paradigm change, giving more consideration to children. When children are still very young, quality of life information is collected using reports from their parents. HRQOL is an important reflection in the understanding of the burden of the child’s living conditions and the evaluation of health care interventions [2, 4]. It is a shared practice for researchers to study children by comparing them in growth and function with typically developed children and every country has its particular normative values [5, 6], including HRQOL and its country specific norms [7].

Researchers have explored the association of children’s quality of life with other individual and social characteristics. According to the gender, no differences have been observed between boys and girls, in a sample of Dutch infants (0–1 years old), toddlers (2–4 years old) and young children (5–7 years old), with the exception of the “problem behaviour” scale of the TNO AZL Preschool Children Quality of Life (TAPQOL) in infants [8]. Moreover, some researchers have found that, as girls become older (from eight to eighteen years old), their perception of HRQOL worsens in comparison to boys [9]. Others have found the opposite, with data showing that, the lower the age of girls, the worse their perception of quality of life (the worst was found in 5–7 years old group) [10]. Finally, other studies report that it is the boys who have worse levels of HRQOL as they become older (from five to twenty years old) [11]. Therefore, the data are very disparate, possibly due to the conditions of each study population and the particularities of each type of disease. On the other hand, lower child HRQOL is linked to parental socioeconomic factors, such as lower levels of parental education, supported by a European multicentre study involving a large sample of children or an Australian longitudinal study (follow-up from four through to age thirteen years) [12, 13]. Moreover, parental education is the strongest risk factor for parent-reported child mental health problems aged 4 to 11 years [14].

HRQOL has proved to be an important construct in the evaluation of the effect of disease management [15].

People with chronic conditions have shown low HRQOL [16]. In addition, the assessment of the child’s physical and psychosocial HRQOL is partly a reflection of the child’s perception toward the emotional state of his or her parents [17]. Thus, the study of HRQOL might facilitate the development of psycho-emotional intervention programmes aimed at parents or caregivers of children when professionals perceive psychological distress associated with the illness or difficulties experienced by their children [18].

HRQOL questionnaires are usually developed for clinical trial settings, but they are not adapted to support the clinical practice [19]. There are different HRQOL questionnaires for use with children: Pediatric Quality of Life Inventory (PedsQL™) [20], KINDL [21], DISABKIDS [22], Infant Toddler Quality of Life Questionnaire (ITQOL) [23], Warwick Child Health and Morbidity Profile (WCHMP) [24], Functional status II (FSII-R) [25], TNO-AZL Preschool Children Quality of Life questionnaire (TAPQOL) [26] or Nordic Quality of Life questionnaire for children [27]. Most of them are parent reports, although, depending on age-appropriateness, some of them also have self-reporting versions. To date, the different validated age-appropriate questionnaires mentioned above measure HRQOL in infants (0–1 years), toddlers (2–4 years), and young children (5–7 years).

The Pediatric Quality of Life Inventory (PedsQL) is a measure developed by Varni et al. [20] which aims to evaluate health-related quality of life in children and adolescents with healthy or acute and chronic health conditions. PedsQL has a generic core module and disease-specific modules. This makes it possible to distinguish between healthy and unhealthy populations or to indicate the severity of the chronic health condition. Thus, this instrument makes it possible to assess and study how the HRQOL of a single case is and where it stands in relation to its peer group, as well as to make comparisons between different groups. Depending on the age of the sample, there is a version for infants (13–24 months), toddlers (ages 2–4), young children (ages 5–7), children (ages 8–12), adolescents (ages 13–18), young adults (18–25) and adults. The PedsQL measures four dimensions: Physical Functioning, Emotional Functioning, Social Functioning and School Functioning. This instrument has been widely used and diverse versions have been validated in different countries, such as Argentina [28], Brazil [29], Korea [30] and China [31], with evidence supporting good psychometric proprieties.

HRQOL instruments are necessary for analysing adults’ and children’s quality of life. As was previously mentioned, it is mostly used with the chronically ill population. We have also seen that each country needs its own norm, in order to compare normative populations to other groups of children with different diseases and conditions. Therefore, it is necessary to validate an updated HRQOL instrument with the Spanish normative children population.

The aim of the current study was to provide Spanish reference data for the PedsQL parent report for toddlers (2–4 years) [20]. In addition, this study adapted and explored the psychometric properties of the PedsQL in this population. The hypotheses were that: (1) the structure of the validated instrument has no variation with respect to the original instrument, and (2) in the HRQOL, parent’s education level shows differences, whereas children’s gender does not.

## Method

### Participants

The minimum sample size was calculated considering the number of children aged 18–36 months in Spain, and the recommendation of having at least between 5 and 10 participants per item of the instrument to be validated [32]. First criteria indicated a sample size with a minimum of 385 participants [33] and second criteria indicated 210 participants. Thus, we considered a minimum sample size of 450 subjects.

A total of 546 parents or legal guardians of infants aged 18 to 36 months were recruited, of whom 478 were selected for the final study. The recruitment of the sample was incidental. Of these 478 parents (Table 1), 89.5% were mothers, parents had an age range of 19 to 61 years (M = 35.26, SD = 5.72), and 69.8% were residents in Andalusia. To be eligible for the study, participants had to be over 18 years old, father, mother or legal guardian of at least one child aged 18 to 36 months, speak Spanish and have no linguistic barriers for filling the instruments, and have answered all the items of the different instruments. The children had a mean age of 26.75 months (SD = 5.88) and 49.6% were female.

**Table 1:** Demographic data of the sample (N = 478)

Parents’ age (N = 468), mean (*SD*)	35.26 (5.72)
Respondent, n (%)	Mother: 428 (89.5) Father: 47 (9.8) Legal guardian: 3 (0.6)
Education level, n (%)	Completed primary education: 15 (3.1) Completed secondary education: 57 (11.9) Completed vocational training: 109 (22.8) Completed high school diploma: 38 (7.9) Completed university: 259 (54.2)
Employment situation, n (%)	Unemployed: 94 (19.7) Half-time job: 125 (26.2) Full-time job: 239 (50) Other situations: 20 (4)
Income, n (%)	< 600 €: 14 (2.9) 600–900 €: 14 (2.9) 900–1200 €: 67 (14) 1200–1800 €: 90 (18.8) 1800–2400 €: 108 (22.6) 2400–3000 €: 87 (18.2) 3000–4500 €: 73 (15.3) > 4500: 25 (5.2)
Number of children, mean (*SD*)	1.53 (0.67)
Children’s age in months, mean (*SD*)	26.75 (5.88)
Sex of children, n (%)	Female: 237 (49.6) Male: 241 (50.4)

## Measures

*Sociodemographic questionnaire ad hoc.* This questionnaire gathered information about the following factors: age, province of residence, education level, current employment status, total household income, mother tongue, language spoken at home, presence of chronic illness and/or disability and the number of children.

*Pediatric Quality of Life Inventory* (PedsQL™ 4.0 or PedsQL) [20]; Spanish translation by Mapi Research Institute (http://www.pedsql.org/translations.html). The general module answered by parents for children aged 2–4 years consists of 21 items, rated on a Likert-type scale from 0 (*never*) to 4 (*almost always*), distributed on four scales: physical health and activities (8 items), emotional state (5 items), social activities (5 items) and school or day-care activities (3 items). The instrument generates different dimensions: physical functioning, emotional functioning, social functioning and school functioning. For the calculation of the HRQOL measure, the items are linearly transformed to a scale of 0-100 (0 = 100, 1 = 75, 2 = 50, 3 = 25, 4 = 0); higher scores indicate better HRQOL. Dimension scores are calculated by dividing the sum of the item scores by the number of items answered. Cronbach’s alpha in the original instrument is 0.90 for the total score, and from 0.75 to 0.88 for the subscales.

*Questionnaire for measuring health-related quality of life in children and adolescents* (Kiddy-KINDL-R or KiddyKINDL) [21, 34]. This questionnaire consists of 24 items distributed in six subscales (four items for each subscale): Physical well-being, emotional well-being, self-esteem, family, friends and nursery school/kindergarten. In a Spanish validation [35], Cronbach’s alpha was 0.90 for the total score, and from 0.64 to 0.80 for the subscales.

### Procedure

Firstly, we contacted Mapi Research Trust (www.mapitrust.org) to request permission to use the instrument and we obtained the licence. The company provided us with the Spanish version of the instrument for parent report for toddlers aged between 2 and 4 years. Incidental sampling was used to recruit from the general population, with the collaboration of kindergarten and nursery schools at the national level. We contacted them through e-mail, phone call or face to face, by sending a letter of request to collaborate and participate, with a link to the questionnaire or a poster with information and a QR code with access to the questionnaire. The data collection period lasted from February to July 2022.

Participants checked the box that corresponded to the informed consent and then completed the instruments anonymously on Google Forms. The indications were that it would take 15 min to complete, and the administration was self-applied and individual. Along with the information, an email address and a phone number were also provided for any questions.

A sample of 7 mothers completed the pilot study to ensure that there were no problems in understanding the questionnaire or recording the responses. A free response box was provided for them to write down any doubts, problems understanding the questionnaires or suggestions.

### Data analysis

The first step of the statistical analysis was to perform a descriptive analysis of the items. The factor structure of the instrument was assessed with a Confirmatory Factor Analysis (CFA), using the diagonally weighted least squares (DWLS) procedure. Chi-square (*χ*²), Comparative Fit Index (CFI), Tucker-Lewis Index (TLI), and Root Mean Square Error of Approximation (RMSEA) with 90% confidence interval were used to analyse the goodness of fit of the model. When *χ*² < 2, CFI > 0.97, TLI > 0.97 and RMSEA < 0.05 [34], the fit was defined as good. When *χ*² < 3, CFI > 0.95, TLI > 0.95 and RMSEA < 0.08, the fit was defined as acceptable [36, 37]. Nevertheless, when the sample size is small (< 500), the use of a more flexible criterion is suggested (CFI > 0.90, TLI > 0.90 and RMSEA < 0.10) [38], thus we considered this last criterion. The internal consistency of the items on the questionnaire and their subscales were evaluated with Cronbach’s alpha. Recorded sociodemographic variables were compared with means of PedsQL™ 4.0 subscales using Mann-Whitney U test and Kruskal-Wallis test. The evidence of validity was analysed with another quality-of-life instrument, using Spearman’s rank correlation coefficient. All the analyses were carried out with IBM SPSS Statistics v.29, IBM SPSS Amos Graphics 23 and RStudio.

## Results

### Descriptive analysis of the items

The results of the descriptive analysis of the items are shown in Table 2. The mean scores on the items ranged between 57.95 (Item 6) and 97.49 (Item 16), and the standard deviation ranged between 8.5 (Item 16) and 27.98 (Item 6). The median skewness was − 1.65 and kurtosis 3.84. The mean score of the total sample (*N* = 478) on the instrument was 83.01 (*SD* = 10.88). All the corrected item-total correlations were above 0.3, except for Item 16 (0.27), which did not discriminate correctly, and no item was removed due to *Cronbach’s alpha if item deleted*.

**Table 2:** Descriptive analysis of items

Item	*M*	*SD*	Skewness	Kurtosis	Corrected item-total correlation	Cronbach’s alpha if item deleted
1	94.04	16.93	-3.21	10.73	0.48	0.84
2	93.25	18.75	-3.32	11.53	0.48	0.84
3	91.68	18.39	-2.46	6.25	0.54	0.83
4	91.16	17.63	-2.33	6	0.55	0.84
5	82.79	22.51	-1.14	0.55	0.32	0.85
6	57.95	27.98	-0.12	-0.58	0.44	0.84
7	79.24	21.21	-0.81	0.38	0.51	0.84
8	77.77	23.10	-0.75	-0.11	0.46	0.84
9	74.84	21.93	-0.53	-0.30	0.35	0.84
10	84.72	17.42	-0.78	-0.31	0.50	0.84
11	59.47	24.83	0.09	-0.63	0.47	0.84
12	64.12	27.65	-0.52	-0.30	0.36	0.85
13	89.70	16.42	-1.51	1.69	0.40	0.84
14	83.73	23.20	-1.48	1.88	0.51	0.84
15	90.06	16.92	-1.77	3.04	0.44	0.84
16	97.49	8.50	-3.58	13.12	0.27	0.85
17	91.63	19.64	-2.90	9.04	0.54	0.83
18	90.48	20.56	-2.60	7.07	0.55	0.83

### Confirmatory factor analysis and internal consistency

Kaiser-Meyer-Olkin (KMO) and Bartlett’s test of sphericity were conducted to evaluate the factorability before Confirmatory Factor Analysis (CFA). The KMO measure of sampling adequacy was 0.84 and the significance of Bartlett’s test of sphericity was 0.00 (*p* < .001).

**Fig. 1:**
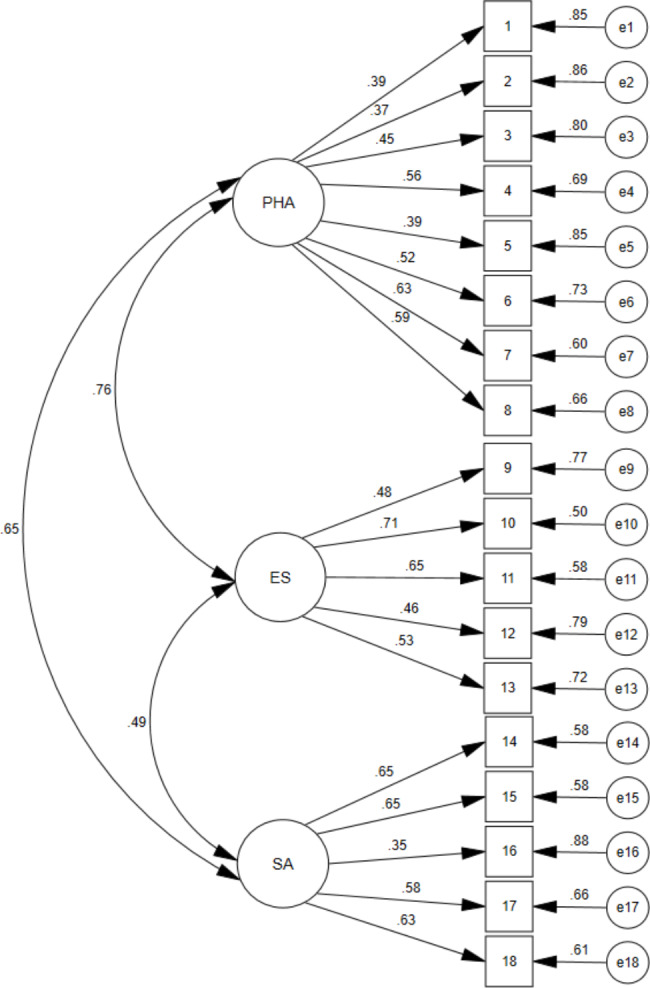
Confirmatory Factor Analysis model. PHA: Physical Health and Activities; ES: Emotional State; SA: Social Activities

The three-factor structure proposed by the authors of the original scale was analysed with a CFA. The model’s fit indices are shown in Table 3. The CFI, TLI and RMSEA had acceptable fit results considering the criteria of Weston and Gore [36]. Figure 1 shows the model’s factor distribution.

**Table 3:** Fit indices

	*χ*²	*df*	CFI	TLI	RMSEA	90% CI (low-high)
Original Factor Model	320.6	132	0.93	0.92	0.06	0.05 − 0.06

The Cronbach’s alpha coefficient was 0.85 for the full scale, and indices of “Physical Health and Activities”, “Emotional State” and “Social Activities” had a Cronbach’s alpha of 0.75, 0.67 and 0.77 respectively. The reliability analysis and all the factors have good internal consistency. “School Functioning” items (19–21) were excluded, since not all the children included in the sample (*N* = 45) attended kindergarten or school and did not answer these items. The Cronbach’s alpha for this factor was low (*α* = 0.65).

### Descriptive analysis of the scale

The mean score of the global PedsQL score was 83.01 (*SD* = 10.88). The mean scores of the factor structure were 83.49 (*SD* = 12.70) for Factor 1, defined as “Physical Health and Activities”, 74.57 (*SD* = 14.49) for Factor 2, defined as “Emotional State”, and 90.68 (*SD* = 13.31) for Factor 3, defined as “Social Activities”.

The Kolmogorov-Smirnov test (K-S test) showed a non-normal distribution (*p* < .001). The mean scores for the PedsQL and the factors were analysed for each of the sociodemographic variables collected (see Table 4). Statistically significant differences were found for these variables in “physical health and activities” related to parents’ education level (*χ²* = 10.94, *p* = .03), “social activities” related to gender (*z* = -2.05, *p* = .04) and total mean of PedsQL related to parents’ education level (*χ²* = 9.64, *p* = .047).

**Table 4:** Correlation between PedsQL total score and its factors according to sociodemographic characteristics

		n	PHA	ES	SA	Total PedsQL mean
Gender	Male	241	237.11	241.42	227.26	235.26
	Female	237	241.93	237.54	251.95	243.81
Parents’ education level	Completed primary	15	131.83	197.47	153.50	141.23
	Completed secondary	57	236.51	203.68	249.22	223.31
	Completed vocational training	109	234.57	249.23	240.84	240.20
	Completed high school diploma	38	231.03	244.30	233.24	235.99
	Completed university	259	249.71	245.02	242.70	248.97

*Note.* PHA: Physical Health and Activities; ES: Emotional State; SA: Social Activities; PedsQL: Pediatric Quality of Life Inventory

### Evidence of validity

Spearman’s rank correlations between factors scores and total PedsQL mean score were analysed with factors scores and total mean of the Kiddy-KINDL-R instrument. All factors and global scores were positively and significantly correlated (see Table 5).

**Table 5:** Spearman’s rank correlational analysis

	PHA	ES	SA	Total PedsQL mean
PW KINDL	0.27**	0.23**	0.12**	0.26**
EW KINDL	0.43**	0.46**	0.31**	0.49**
SE KINDL	0.37**	0.33**	0.39**	0.44**
FA KINDL	0.31**	0.38**	0.25**	0.36**
FR KINDL	0.37**	0.30**	0.48**	0.46**
SC KINDL	0.41**	0.29**	0.41**	0.46**
Total KiddyKINDL mean	0.49**	0.45**	0.44**	0.57**

*Note*. ** *p* < .01. PHA: Physical Health and Activities; ES: Emotional State; SA: Social Activities; PedsQL: Pediatric Quality of Life Inventory; PW KINDL: Physical Well-being of KiddyKINDL; EW KINDL: Emotional Well-being of KiddyKINDL; SE KINDL: Self-esteem of KiddyKINDL; FA KINDL: Family of KiddyKINDL; FR KINDL: Friends of KiddyKINDL: SC KINDL: School of KiddyKINDL.

### Score distribution

Finally, a percentile table was generated for the scores obtained in this sample (Table 6), in order to guide the normative interpretation of the PedsQL scale scores when deemed appropriate. As noted, higher scores indicated greater HRQOL. In the present sample, total scores of 77.78, 84.72 and 90.28 corresponded to the first, second and third quartiles, respectively.

**Table 6:** Percentiles of the PedsQL scores

Percentiles	PHA	ES	SA	Total PedsQL mean
10	68.75	55.00	75.00	70.69
20	75.00	60.00	85.00	75.00
30	78.13	65.00	90.00	79.17
40	81.25	70.00	90.00	81.94
50	84.37	75.00	95.00	84.72
60	87.50	80.00	100.00	87.50
70	90.63	85.00	100.00	88.89
80	93.75	90.00	100.00	91.94
90	96.88	95.00	100.00	94.58

*Note*. PHA: Physical Health and Activities; ES: Emotional State; SA: Social Activities; PedsQL: Pediatric Quality of Life Inventory

## Discussion

The aim of this study was to provide Spanish reference data about health-related quality of life (HRQOL) in toddlers, that is, children aged between 18 and 36 months. The general module of the Pediatric Quality of Life Inventory parent report for toddlers (2–4 years) (PedsQL™ 4.0) [20] assesses health-related quality of life. Better HRQOL is associated with high scores on this measure. Overall, this sample showed high scores on health-related quality of life. This work has the advantage of providing current descriptive data for a Spanish sample of very young children. This population is difficult to reach because it is a small age group, and many of them are not yet in school. By not considering items related to school, the scale makes it possible to measure the quality of life of all Spanish children of that age, regardless of whether they are in or out of school. This version covers generic aspects in children’s quality of life. Moreover, not only the mean and SD scores are provided, but also the distribution in percentiles for the total score of the scale, as well as for each of the three subscales, allowing for a better understanding of how the scores are distributed among the total number of participants. So, in addition to a total quality of life score, data is provided for the three dimensions: Physical Health and Activities”, “Emotional State” and “Social Activities” from the parents’ point of view.

The first hypothesis of this study was partially confirmed. The confirmatory factor analysis of the original factor model presented acceptable fit with the main indices. The global instrument worked better after excluding the “school functioning” items in this validation, as in the validation conducted in Argentina [28]. Moreover, all parents, regardless of whether their children go to nursery school or not, can fill it in. The results showed adequate psychometric properties. The Spanish PedsQL version had good reliability, although it is lower compared to other validation studies [28, 39, 40]. For scoring purposes, this version may work better if school items were not taken into consideration in this group.

The second hypothesis was also partially confirmed. Mean scores showed no gender differences on the global scale and its subscales, except in social activities, where girls presented more positive scores. These data support those of Schepers et al. [8], who found no differences between infant boys and girls, except that girls showed fewer behavioural problems than boys. On the other hand, results consistent with those obtained here have been found in older children. This is the case of a study of Michel et al. [9], where 8-year-old girls and boys had a similar average score, but the score was decreasing for both in older ages. In this study, at the age of eight years, girls had a significant higher mean score in “peers and social support”, which was also observed in the children’s ages of this sample. Finally, a study found that, in children aged 8–11 years, low parent education level was associated with low quality of life [41], coinciding with the results obtained in this study. In addition, the validity analysis found that there is significant correlation between two different scales that measure health-related quality of life. The global scores of the scales had the highest correlation. Moreover, the “emotional well-being” subscale showed a strong correlation with “physical and emotional health”, as well as the “friends” subscale with “social activities”.

Furthermore, percentiles of the PedsQL subscales and total mean scores were presented for a population of Spanish children in order to provide reference data. The percentile table allows comparing an individual’s scores with a standard range or percentile in the Spanish population. To our knowledge, there are no published studies on reference values of the PedsQL for the Spanish population of this age, although these values are important to facilitate its applicability. As in most measures of perceived health, the total and subdimension scores of the PedsQL should not be interpreted in isolation, but in comparison with the distribution of scores of a reference group. HRQOL studies in adults have demonstrated the usefulness of having reference values obtained from representative samples of the population. Comparisons with the population distribution can be used to detect the HRQOL needs of children whose scores are in the lower percentiles, as well as to evaluate health and social interventions.

## Conclusion

This study does not lack limitations. Our sample was mostly constituted by mothers, and thus it did not adequately reflect the perception of the fathers toward their children. The questionnaire was self-applied and in an online format, thus the error of variance or the response interference could be affected. The number of clinical participants in this sample was not enough to make comparisons with the healthy participants. In future studies, percentile scores will facilitate the analyses across clinical and non-clinical groups of Spanish children.

This study presents a validated instrument for the Spanish population to assess quality of life in children aged 1.5 to 3 years. To our knowledge, this is the first study to examine the quality of life with this measure in a representative group of Spanish parents with toddlers. In conclusion, this study provides Spanish reference data about HRQOL. It shows a healthy sample with a very good quality of life. This instrument has acceptable consistency indices both in the global scale and in the subscales. In addition, it shows good fit indices in the factorial structure. Specifically, PedsQL™ 4.0 evaluates the quality of life related to physical health and activities, emotional state and social activities of toddlers from a parental perspective. This instrument can be useful not only at the individual level to assess the quality of life of a particular child in relation to his or her group thanks to the percentiles, or to make comparisons between different groups, but also to measure the effectiveness of a possible intervention with a pre- and post-test.

## Data Availability

The data that support the findings of this study are available on request from the corresponding author.

## Abbreviations

CFA: Confirmatory Factor Analysis
CFI: Comparative Fit Index
DWLS: Diagonally weighted least squares
ES: Emotional State
EW KINDL: Emotional Well-being of KiddyKINDL
FA KINDL: Family of KiddyKINDL
FR KINDL: Friends of KiddyKINDL
FSII-R: Functional status II
HRQOL: Health-related quality of life
ITQOL: Infant Toddler Quality of Life Questionnaire
Kiddy-KINDL-R or KiddyKINDL: Questionnaire for measuring health-related quality of life in children and adolescents
KMO: Kaiser-Meyer-Olkin
K-S test: Kolmogorov-Smirnov
PedsQL: Pediatric Quality of Life Inventory
PHA: Physical Health and Activities
PW KINDL: Physical Well-being of KiddyKINDL
RMSEA: Root Mean Square Error of Approximation
SA: Social Activities
SC KINDL: School of KiddyKINDL
SD: Standard Deviation
SE KINDL: Self-esteem of KiddyKINDL
TAPQOL: TNO AZL Preschool Children Quality of Life
TLI: Tucker-Lewis Index
WCHMP: Warwick Child Health and Morbidity Profile
